# Development and validation of a parsimonious prediction model for positive urine cultures in outpatient visits

**DOI:** 10.1371/journal.pdig.0000306

**Published:** 2023-11-01

**Authors:** Ghadeer O. Ghosheh, Terrence Lee St John, Pengyu Wang, Vee Nis Ling, Lelan R. Orquiola, Nasir Hayat, Farah E. Shamout, Y. Zaki Almallah

**Affiliations:** 1 NYU Abu Dhabi, Abu Dhabi, The United Arab Emirates; 2 Cleveland Clinic Abu Dhabi, Abu Dhabi, The United Arab Emirates; Ben-Gurion University of the Negev, ISRAEL

## Abstract

Urine culture is often considered the gold standard for detecting the presence of bacteria in the urine. Since culture is expensive and often requires 24-48 hours, clinicians often rely on urine dipstick test, which is considerably cheaper than culture and provides instant results. Despite its ease of use, urine dipstick test may lack sensitivity and specificity. In this paper, we use a real-world dataset consisting of 17,572 outpatient encounters who underwent urine cultures, collected between 2015 and 2021 at a large multi-specialty hospital in Abu Dhabi, United Arab Emirates. We develop and evaluate a simple parsimonious prediction model for positive urine cultures based on a minimal input set of ten features selected from the patient’s presenting vital signs, history, and dipstick results. In a test set of 5,339 encounters, the parsimonious model achieves an area under the receiver operating characteristic curve (AUROC) of 0.828 (95% CI: 0.810-0.844) for predicting a bacterial count ≥ 10^5^ CFU/ml, outperforming a model that uses dipstick features only that achieves an AUROC of 0.786 (95% CI: 0.769-0.806). Our proposed model can be easily deployed at point-of-care, highlighting its value in improving the efficiency of clinical workflows, especially in low-resource settings.

## Author summary

Urine culture tests are often ordered to help early detection of bacteria in the urine in various clinical settings. Notwithstanding their importance in clinical decision-making, urine culture tests add cost and burden on medical staff as they require a long waiting time. In this work, we propose a low-cost machine learning model to provide real-time predictions of urine culture results at point-of-care. The proposed approach is based on a simple model that requires a minimal feature set, making it easy to implement in real-clinical settings. By developing and validating the model on real-world outpatient data from Abu Dhabi, we found that our model outperformed the clinical baselines. Our findings underscore the potential of machine learning models in optimizing clinical workflow efficiency by providing timely predictions.

## Introduction

Urine cultures have long been used for detecting the presence of specific microorganisms in the urine. It is usually ordered for patients with urinary symptoms mainly to evaluate for the presence of bacteria in urine. A positive urine culture result is considered the gold standard in the diagnosis and treatment of certain infections, such as urinary tract infection (UTI) [1, 2]. Despite their prevalence, urine cultures are not always necessary and diagnostic stewardship seeks best practices for ordering such tests [3]. The process of obtaining the results of a urine culture test is also time-consuming, and it relies on the examiners’ experience, which may not always be readily available.

Urine dipstick test is a point of care (POC) test where a strip treated with chemicals is dipped in a urine sample. The strip then changes color to indicate the concentration of certain substances [4]. Although popular and easy to use, disptick tests tend to lack sensitivity and specificity, which limits their optimal use for predicting urine culture results in clinical practice [5]. Considering the costs associated with processing a urine culture test, there is a prominent need for a predictive model at POC that can assist clinicians in their decision-making process.

Several existing studies investigated the prediction of urine culture results, and most approaches rely on using urinalysis results as predictive variables. For example, [6] use the results of an automated urinalysis system to build a model that predicts urine culture results in a cohort of inpatients and outpatients. Another example is by [7], where the authors build a system for predicting urine culture results from urine flow cytometry in a large cohort of emergency encounters. While useful, most of these models rely on data collected using specific technologies for urinalysis that may not always be available at different clinical institutions. While previous work focus on the prediction of urine culture results in the emergency department [7] or across a general cohort of inpatient and outpatient encounters [6], many urine cultures take place in the outpatient setting, such as in primary care or elective encounters where a clinical decision is often made at POC. Additionally, previous work does not investigate the use of other readily available information, such as previous disease and procedures, patient demographics, and comorbidities, which can be augmented with dipstick results for the prediction of urine culture results.

**Fig 1:**
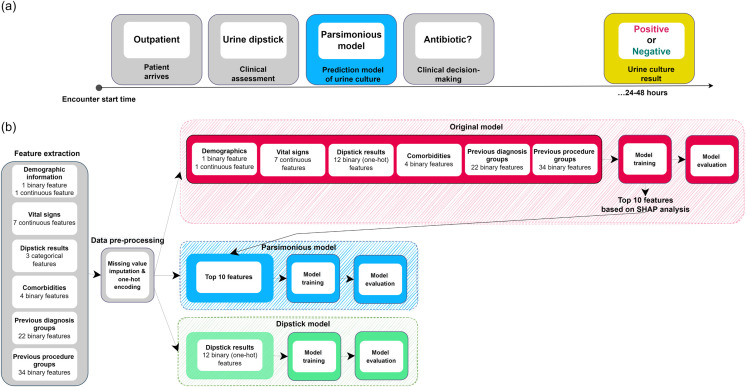
Overview of the proposed model. (a) In this figure, we illustrate an example of an outpatient encounter. After evaluating the patient’s symptoms, a clinician may perform a urine dipstick test while they wait for the urine culture results. Our proposed parsimonious model can make a prediction ahead of the culture results to inform the decision-making process. (b) In this figure, we summarize the model development process. We first extract the features, pre-process the data, and then develop three prediction models with all the features (original model), with the top ten predictive features (parsimonious model), and with the dipstick features only (dipstick model).

To this end, we develop a machine learning-based parsimonious model for the prediction of positive urine culture results in outpatient visits. Our proposed model can predict the result of the urine culture based on a minimal feature set of dipstick results and readily available information in the electronic patient record. We train and evaluate the model using observational retrospective data collected at Cleveland Clinic Abu Dhabi (CCAD) in the United Arab Emirates (UAE). Our data-driven approach of selecting a minimal feature set demonstrates significant improvements in predicting urine culture results when compared to using dipstick results alone, demonstrating its potential in supporting decision making at POC in outpatient settings without increasing the burden on the staff. An overview of use case and the model development and evaluation pipeline is shown in Fig 1. To allow for reproducibility and external validation of our proposed work, we made our code available at https://github.com/nyuad-cai/Parsimonious-Model-PUC.

## Materials and methods

### Dataset

We retrieved anonymized data collected between March 2015 and March 2021 at CCAD, which is a multi-specialty large hospital with primary, secondary, and tertiary care facilities in Abu Dhabi, UAE. This retrospective study was approved by the Institutional Review Board of CCAD (Ref: A-2019-054) and NYU Abu Dhabi (Ref: HRPP-2020-173). Informed consent was not required as the study was determined to be exempt. We report the study in accordance to the Transparent reporting of a multi-variable prediction model for individual prognosis or diagnosis (TRIPOD) guidance [8]. The checklist is shown in S1 File.

To define the patient cohort, we designed an inclusion and exclusion criteria in collaboration with clinical experts. We include outpatient encounters only and exclude all other encounters that represent in-patient admissions. The outpatient setting at the dataset’s institution spans primary, secondary and tertiary care. Since the study focuses on adult patients, we exclude encounters of patients who were less than 18 years old at the time of the start of the encounter. We also only include encounters associated with a urine culture, as we use the urine culture result to define the model’s output. Finally, we perform a temporal patient split to obtain a training set of encounters recorded between 2015 and 2019, and a test set of encounters recorded between 2020 and 2021. We use the training set for model development and the test set for model evaluation. All of the results are reported on the test set.

### Input features

#### Demographics & vital-sign measurements

To define the input features of the model, we first extract data that is collected at the beginning of each encounter: demographic information and vital-sign measurements. The demographic features included patient age (numerical) and biological sex (binary). The vital-sign measurements are all numerical and include six variables: pulse, respiratory rate, oxygen saturation, temperature, systolic blood pressure, and diastolic blood pressure. If a vital-sign measurement is missing, we perform mean imputation.

#### Patient history

First, we define and extract four binary features to explicitly represent patient comorbidities: cancer, diabetes, hypertension, and hyperlipidemia, where 1 indicates the presence of the comorbidity and 0 otherwise. Cancer was explicitly recorded as a binary feature in the patient encounter data. We extract the three other conditions for each encounter using International Classification of Diseases (ICD)-10 codes recorded in any of the patient’s previous encounters, which could be outpatient or otherwise. The ICD-10 codes are summarized in S2 File.

Next, we extract the patient’s history of disease using all of the ICD-10 codes recorded in any previous encounter. We group the ICD-10 codes based on the high-level categorization of the type of disease [9], resulting with the 22 binary features. Similarly, we group history of previous procedures according to custom hospital codes, where each group indicates the type of procedure. This process results with 34 binary features each representing a unique procedure group. If a patient does not have previous encounters at the hospital, we set all of the patient history features to 0.

#### Urine dipstick results

For each encounter in our dataset, we extract any associated urine dipstick results collected within the same encounter. Based on clinical expertise and clinical literature [1, 10–12], we identified three substances of interest as input features to our model: nitrites, leukocyte esterase, and hemoglobin. We then clean the data by resolving spelling mistakes and inconsistencies. Missing values are replaced with results of microscopic urinalysis, if available within the same encounter. We apply one-hot encoding to the final categorical features, except for nitrite which we consider as a binary feature (positive/negative). Encounters with no record of urine dipstick or microscopic analysis are assigned with the most frequent value in the training set for each respective feature. We report the statistical distribution of all input features, including mean and standard deviation for the numerical features and a distribution count for categorical features.

### Ground-truth labels

The goal of our model is to predict whether a urine culture is likely to grow bacterial agents [1]. To this end, we process the urine culture results to define the ground-truth labels. Each urine culture result is associated with the time of sample collection, result time, and semi-structured text summarizing the culture result of the sample. Positive samples are typically described through the explicit mention of a significant growth of a bacterial agent [13]. The description may also indicate the quantity of Colony Forming Units per milliliter (CFU/ml). International guidelines use varying thresholds to confirm a diagnosis [14–16]. Hence, we define two labels to represent a positive urine culture: ≥ 10^4^ CFU/ml and ≥ 10^5^ CFU/ml, with the latter being more definitive and the primary outcome of this work. If there is no significant growth of bacteria, we assume that the culture is negative. Each encounter eventually has two binary output labels, one for each bacterial count threshold.

### Predictive modeling

#### Model development

We develop three multivariable logistic regression models for each output label. The motivation behind using a multivariable logistic regression is its relative simplicity and often comparative performance to other more complex machine learning models, all of which facilitates easy deployment at POC [17–20]. The first model, defined as the “original model”, processes all of the input features. We then perform SHapley Additive exPlanations (SHAP) analysis to identify the top ten features for the parsimonious model [21]. SHAP values are based on a game theory approach for calculating each feature’s contributions to the final model prediction [22]. The SHAP value for each feature is indicative of the relative importance of the input variables and its impact on the predictions. While most commonly used as a model interpretability method, SHAP values can be used as a feature-selection methodology to identify the most predictive features [23]. To measure the importance of each feature we use the mean absolute SHAP value across the overall population.

Using the ten most predictive features identified by the original model, we then train a new model for each output label, which we refer to as the parsimonious model. The parsimonious model is driven by the need for low-cost models that can be easily deployed in practice [24]. As a clinical baseline, we train another set of models using urine dipstick features only. We consider this model as a strong clinical baseline since previous work highlighted the usefulness of dipstick results in predicting urine culture outcomes [1].

To train all of the described models, we perform 5-fold cross validation randomized hyperparameter search on the training set. The hyperparameters include type of penalty, regularization strength, optimizer, and maximum number of iterations, and the search ranges are listed in S3 File. We select the best hyperparameters based on the highest average cross-validation performance, which are then used to fit the final models.

#### Model evaluation

We evaluate the final models on the test set in terms of the Area Under the Receiver Operating characteristic Curve (AUROC) and the Area Under the Precision-Recall Curve (AUPRC) and visualize associated curves. The AUROC summarizes the model’s ability in discriminating between positive and negative samples [25], while the AUPRC illustrates its performance considering class imbalance [26]. We also report model calibration in terms of calibration slope and intercept. Calibration is a reflection of how well the model’s probability predictions reflect the true distribution of the ground-truth labels [27, 28]. We assess model performance across the overall population, females and males, and two age groups. All results are reported with confidence intervals computed using bootstrapping with 1,000 iterations [29]. We perform all experiments using Python (version 3.7.3) and scikit-learn (version 1.1.1).

## Results

### Patient cohort

**Fig 2:**
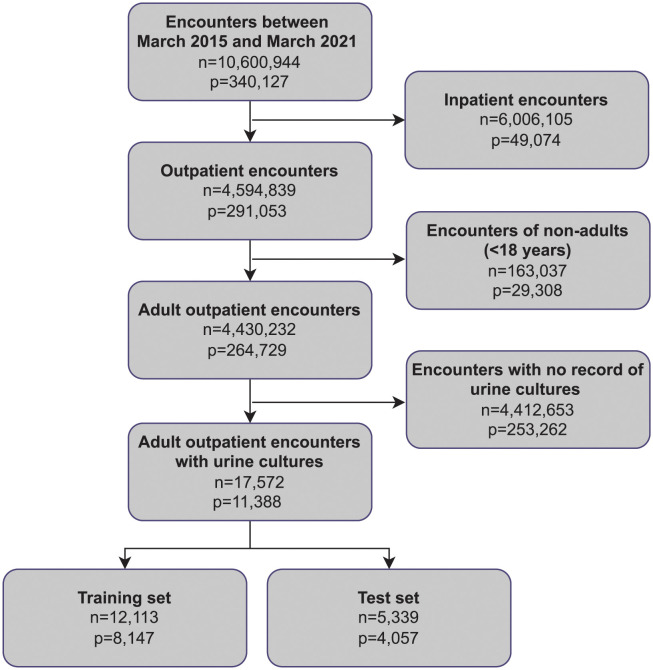
Flowchart of inclusion and exclusion criteria. We apply the inclusion and exclusion criteria to obtain the training set, which we use for model development, and the test set, which we use for model evaluation. In the figure, n represents the number of unique encounters and p represents the number of unique patients since a unique patient could have multiple encounters.

The results of applying the inclusion and exclusion criteria are shown in Fig 2. The final training set consists of 12,113 unique encounters and 8,147 unique patients, while the final test set consists of 5,339 unique encounters and 4,057 unique patients. In Table 1, we summarize the characteristics of the patient cohort. We observe that the distribution of age and sex is similar across the training and test sets, with a mean age of 49.1 ± 17.6 years and 58.8% females in the training set, and a mean age of 49.2 ± 17.0 years and 50.0% females in the test set. The prevalence of positive urine cultures based on the ≥ 10^5^ CFU/mL threshold was 13.7% and 14.4% in the training and and test sets, respectively. We also observe a higher incidence of positive urine cultures in females than in males, 9.7% vs 4.0% in the training set and 10.5% vs 4.0% in the test set. Similarly, a higher incidence is observed among the older population, 10.2% vs 3.5% in the training set and 10.9% vs 3.6% in the test set. In Table 2 we summarize the distributions of the demographic features, vital-sign measurements, comorbidities and dipstick results. The distribution of the other patient history features is summarized in Table 3.

**Table 1:** Summary of patient cohort. We describe the characteristics of the patient cohort across the training and test sets. Here, n represents number, std represents standard deviation, and % is percentage. We also report the distribution of ground-truth labels across patient subgroups.

Characteristic	Training set	Test set
Outpatient encounters (n)	12,113	5,339
Unique patients (n)	8,147	4,057
Age, mean (std)	49.1 (17.7)	49.16 (17.0)
Female, n (%)	7,123 (58.8)	2,670 (50.0)
**Positive urine culture, n (%)**		
≥ 10^5^ CFU/mL		
Overall population	1,662 (13.7)	769 (14.4)
Females	1,184 (9.7)	558 (10.5)
Males	478 (4.0)	211 (4.0)
< 40 years old	432 (3.6)	187 (3.5)
≥ 40 years old	1,230 (10.2)	582 (10.9)

**Table 2:** Overview of input features. Summary of the model input features across the training and test sets, where mean and standard deviation (std) are shown for numerical features, and the number (n) and percentage (%) are shown for categorical features, such as comorbidities and urine dipstick features.

Input feature	Training set	Test set
**Demographics**		
Age, mean (std)	49.1 (17.7)	49.2 (17.0)
Female, n (%)	7,123 (58.8)	2,670 (50.0)
**Comorbidities, n (%)**		
Diabetes	1,608 (13.3)	853 (16.0)
Hypertension	1,655 (13.7)	1,012 (19.0)
Hyperlipidemia	208 (1.7)	299 (5.6)
Cancer	689 (5.7)	993 (5.5)
**Vital sign, unit, mean (std)**		
Respiratory rate, *breaths per minute*	17.6 (3.2)	17.4 (3.9)
Not Recorded	8133 (67.1)	3566 (66.8)
Pulse rate, *beats per minute*	78.5 (12.6)	78.1 (12.3)
Not Recorded	7674 (63.4)	3497 (65.5)
Oxygen saturation, *%*	99.5 (2.4)	99.3 (2.8)
Not Recorded	8133 (64.7)	3518 (65.9)
Temperature auxiliary, °*C*	36.5 (0.8)	36.7 (0.9)
Not Recorded	8268 (68.3)	3760 (70.4)
Systolic blood pressure, *mmHg*	127.9 (16.8)	127.3 (15.9)
Not Recorded	7577 (62.6)	3483 (65.2)
Diastolic blood pressure, *mmHg*	72.7 (12.1)	75.3 (11.1)
Not Recorded	7577 (62.6)	3483 (65.2)
**Urine dipstick, n (%)**		
Nitrite		
Negative	8,624 (71.2)	4,304 (80.6)
Positive	741 (6.1)	300 (5.6)
Not Recorded	2,748 (22.7)	735 (13.8)
Leukocyte esterase		
3+	1,092 (9.0)	511 (9.6)
2+	851 (7.0)	383 (7.2)
1+	1,024 (8.5)	480 (9.0)
Trace	988 (8.2)	325 (6.0)
Negative	5,409 (44.7)	2,905 (54.4)
Not Recorded	2,749 (22.7)	735 (13.8)
Hemoglobin		
4+	102 (0.8)	240 (4.5)
3+	621 (5.1)	227 (4.3)
2+	516 (4.3)	281 (5.3)
1+	906 (7.5)	472 (8.8)
Trace	1,997 (16.5)	814 (15.3)
Negative	5,223 (43.1)	2,570 (48.1)
Not Recorded	2,748 (22.7)	735 (13.8)

**Table 3:** Summary of the ICD code groups and procedure code groups used as input features to train the models, in terms of count (encounters) and percentage in both the training and test sets, respectively.

Features, n (%)	Training set	Test set
**ICD diagnosis code groups**		
Certain infections and parasitic diseases	1411 (11.7)	993 (18.6)
Neoplasms	9030 (74.6)	4452 (83.4)
Diseases of the blood and blood-forming organs	9030 (74.6)	4452 (83.4)
Endocrine, nutritional and metabolic diseases	2866 (23.7)	1745 (32.7)
Mental, Behavioral and Neurodevelopmental disorders	414 (3.4)	326 (6.1)
Diseases of the nervous system	1501 (12.4)	1129 (21.2)
Diseases of the eye and adnexa	9030 (74.6)	4452 (83.4)
Diseases of the ear and mastoid process	9030 (74.6)	4452 (83.4)
Diseases of the circulatory system	2818 (23.3)	1615 (30.3)
Diseases of the respiratory system	1646 (12.6)	1017 (19.1)
Diseases of the digestive system	2880 (23.8)	1866 (35.0)
Diseases of the skin and subcutaneous tissue	979 (8.1)	735 (13.8)
Diseases of the musculoskeletal system and connective tissue	2628 (21.7)	1538 (28.8)
Diseases of the genitourinary system	3670 (30.3)	2132 (39.9)
Pregnancy, childbirth, and puerperium	222 (1.8)	118 (2.2)
Certain conditions originating in the perinatal period	2 (0.0)	5 (0.1)
Congenital malformations, deformations and chromosomal abnormalities	340 (2.8)	166 (3.1)
Symptoms, signs, and abnormal clinical laboratory findings	5523 (45.6)	3019 (56.6)
Injury, poisoning, and certain other consequences of external causes	807 (6.7)	590 (11.1)
Codes for special purposes	0 (0.0)	17 (0.3)
External causes of morbidity	10 (0.1)	1 (0.0)
Factors influencing health status and contact with health services	4591 (37.9)	2801 (52.5)
**Procedure code groups**		
Lab chemistry orderables	5679 (46.9)	3339 (62.5)
Urine orderables	4557 (37.6)	2099 (39.3)
Lab blood orderables	6576 (54.3)	3365 (63.0)
Microbiology—general orderables	2813 (23.2)	1654 (31.0)
Poct orderables—device	2310 (19.1)	2047 (38.3)
Procedure/minor surgical orderables	898 (7.4)	550 (10.3)
Pathology/cytology orderables	832 (6.9)	467 (8.8)
IMG US orderables	3062 (25.3)	1895 (35.5)
CV ECG / EKG orderables	2892 (23.9)	1660 (31.1)
Genetic testing	356 (2.9)	214 (4.0)
HLA lab orderables	358 (3.0)	302 (5.7)
Blood bank test orderables	323 (2.7)	204 (3.8)
IMG CT orderables	3003 (23.8)	1785 (33.4)
IMG diagnostic imaging/x-ray orderables	4325 (35.7)	2292 (42.9)
IMG MRI orderables	1228 (10.1)	833 (15.6)
Body fluids and stools orderables	195 (1.6)	131 (2.5)
ADT orderables	839 (6.9)	565 (10.6)
Visit typelinked ref orders	0 (0.0)	263 (4.9)
Ophthalmology services orderables	68 (0.6)	56 (1.1)
Core measures orderables	6 (0.1)	1 (0.0)
General surgical orderables	217 (1.8)	105 (2.0)
GI procedure orderables	255 (2.1)	244 (4.6)
IMG dexa orderables	172 (1.4)	122 (2.3)
Ophthalmology zeiss orderables	2393 (19.8)	1377 (25.8)
IMG fluoroscopy orderables	592 (4.9)	373 (7.0)
CV echo orderables	572 (4.7)	334 (6.3)
ONCBCN communication	7 (0.1)	6 (0.1)
Blood bank product orderables	99 (0.8)	66 (1.2)
PFT orderables	1223 (10.1)	672 (12.6)
Outpatient referral orderables	119 (1.0)	91 (1.7)
IMG NM orderables	329 (2.7)	250 (4.7)
Neurology orderables	96 (0.8)	77 (1.4)
IMG mammography orderables	15 (0.1)	50 (0.9)
ENT orderables	23 (0.2)	8 (0.2)

### Performance evaluation

**Fig 3:**
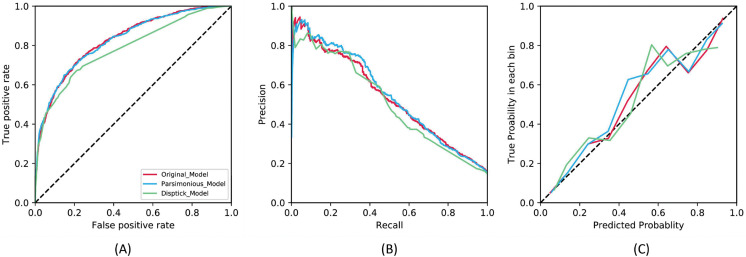
Performance curves on the test set. Fig 3A Receiver operating characteristic curves, Fig 3B precision-recall curves, and Fig 3C calibration curves are shown for the original, parsimonious and dipstick models for the ground-truth label ≥ 10^5^ CFU/ml.

We compare the performance of the best original model with all of the input features, parsimonious model with the top ten features identified by the former via SHAP analysis, and the dipstick only model. The performance results on the test set for ≥ 10^5^ CFU/ml is visualized in Fig 3 for the Fig 3A receiver operating characteristic curve, Fig 3B, precision-recall curve, and Fig 3C calibration curves. In Table 4, we summarize all the metrics with 95% confidence intervals.

**Table 4:** Performance evaluation results on the test set. We report the performance results for the area under the receiver operating characteristic curve (AUROC), area under the precision recall curve (AUPRC), and calibration slope and intercept. The results are shown for the overall population and patient sub-groups. All results are reported with 95% confidence intervals computed using bootstrapping with 1,000 iterations [29].

Population	Result	Original model	Parsimonious model	Dipstick model
Overall population	AUROC	0.831 (0.816, 0.846)	0.828 (0.810, 0.844)	0.786 (0.769, 0.806)
	AUPRC	0.542 (0.508, 0.578)	0.550 (0.511, 0.593)	0.484 (0.445, 0.522)
	Calibration slope	0.951 (0.870, 1.028)	0.951 (0.864, 1.028)	0.906 (0.773, 1.022)
	Calibration intercept	0.045 (0.016, 0.077)	0.063 (0.031, 0.097)	0.069 (0.014, 0.124)
Females	AUROC	0.769 (0.743, 0.792)	0.767 (0.743, 0.792)	0.760 (0.738, 0.783)
	AUPRC	0.558 (0.515, 0.599)	0.575 (0.533, 0.617)	0.533 (0.496, 0.579)
	Calibration slope	0.928 (0.825, 1.022)	0.938 (0.840, 1.035)	0.925 (0.788, 1.052)
	Calibration intercept	0.057 (0.020, 0.095)	0.080 (0.042, 0.120)	0.119 (0.045, 0.184)
Males	AUROC	0.875 (0.85, 0.896)	0.868 (0.841, 0.892)	0.806 (0.775, 0.836)
	AUPRC	0.505 (0.434, 0.57)	0.486 (0.416, 0.555)	0.409 (0.345, 0.491)
	Calibration slope	0.995 (0.869, 1.110)	0.951 (0.819, 1.065)	0.805 (0.538, 1.014)
	Calibration intercept	0.033 (-0.021, 0.086)	0.015 (-0.046, 0.085)	-0.001 (-0.088, 0.112)
< 40 years old	AUROC	0.809 (0.777, 0.842)	0.802 (0.768, 0.836)	0.749 (0.707, 0.788)
	AUPRC	0.407 (0.342, 0.485)	0.424 (0.357, 0.503)	0.373 (0.306, 0.456)
	Calibration slope	0.881 (0.654, 1.026)	0.798 (0.466, 1.048)	0.754 (0.495, 0.998)
	Calibration intercept	0.063 (0.004, 0.139)	0.082 (-0.009, 0.191)	0.063 (-0.029, 0.161)
≥ 40 years old	AUROC	0.837 (0.819, 0.855)	0.837 (0.821, 0.854)	0.800 (0.780, 0.819)
	AUPRC	0.583 (0.542, 0.625)	0.588 (0.546, 0.632)	0.533 (0.492, 0.573)
	Calibration slope	0.981 (0.890, 1.062)	0.985 (0.900, 1.068)	0.978 (0.852, 1.071)
	Calibration intercept	0.038 (0.006, 0.070)	0.054 (0.019, 0.087)	0.067 (0.002, 0.141)

Using the ≥ 10^5^ CFU/ml threshold, the original model achieves the best performance across all patient subgroups, with 0.831 (0.816, 0.846) AUROC and 0.542 (0.508, 0.578) AUPRC. The parsimonious model achieves comparable results to the original model with only ten features in the overall population, with 0.828 (0.810, 0.844) AUROC and 0.550 (0.511, 0.593) AUPRC. On the other hand, the worst performing model is the dipstick only model with 0.786 (0.769, 0.806) AUROC and 0.484 (0.445, 0.522) AUPRC. Across both labels in the overall population, we note that all models were well-calibrated, with the slope ranging between 0.906 and 0.951 and intercepts between 0.045 and 0.069, as visualized in the calibration curves in Fig 3C. We include all the results of the model trained using the 10^4^ in S4 File.

When comparing the performance across the female and male patient subgroups, we observe that all models achieved a higher AUROC across males, but a higher AUPRC across females. For example, the parsimonious model achieves a 0.767 AUROC across the female subgroup, compared to 0.868 AUROC across the male subgroup. This implies that the model can better discriminate between the positive and negative classes in the male subgroup. On the other hand, the parsimonious model achieves a 0.575 AUPRC across the female subgroup compared to 0.486 AURPC across the male subgroup for the ≥ 10^5^ CFU/ml label, which is related to the difference in class imbalance across the two subgroups. We also compare the performance of the models across two age subgroups: < 40 and ≥ 40 years old. We note that the models had a comparable performance across the two populations.

We also conduct a subgroup analysis for encounters with a recorded UTI ICD code in the test set, which is equivalent to 137 encounters. In this subgroup, the model achieves an AUROC of 0.806 (0.714, 0.882 95% CI) and AUPRC of 0.587 (0.432, 0.760 95% CI).

### Feature importance

**Fig 4:**
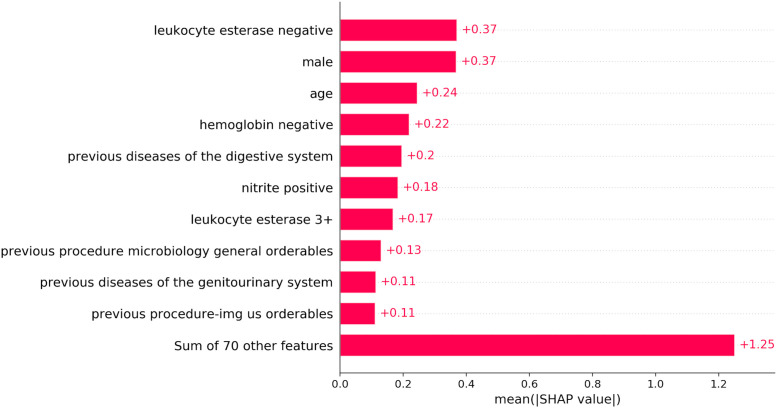
Post-hoc feature importance for the original model. A bar plot showing the mean SHAP value assigned to each input feature.

The top ten predictive features of the original model that were used to develop the parsimonious model are shown in Fig 4 with their mean absolute SHAP values, which indicate their importance with respect to the model’s prediction. For the ≥ 10^5^ CFU/ml label, the top ten features are: negative leukocyte esterase dipstick finding, patient sex, patient age, negative hemoglobin dipstick finding, previous diseases of the digestive system, positive nitrite dipstick finding, +3 leukocyte esterase dipstick finding, previous microbiology procedure, previous diseases of the genitourinary system, and previous ultrasound procedures. Similarly for the ≥ 10^4^ CFU/ml label, the top ten features include previous urine orderables but excluded previous ultrasound procedures. The full list of features and their corresponding SHAP values are shown in S5 File. The final coefficients and intercept of the multivariable logistic regression models in the parsimonious setting are shown in S6 File. We also conducted an analysis where we varied the number of included features in the parsimonious model and observed that by using 10 features the model maintained a comparable performance to that of the original model trained using the full feature set. The results for this analysis are presented in S7 File. To understand how the predictions apply on the patient level, we show the shap analysis for an example encounter in S8 File.

## Discussion

The main contributions of this study is that we propose, develop, and implement a data-driven framework for predicting urine cultures in outpatient visits and evaluate it using a real-world dataset. We specifically focus on the development of a low-cost parsimonious model that can be easily used at POC. We used a dataset collected at a large multi-specialty hospital in Abu Dhabi, UAE. Using ten features only, the parsimonious model achieves a 0.828 AUROC in the overall population for the ≥ 10^5^ CFU/ml label, which is a commonly used threshold in the guidance of clinical decision-making [1, 30]. To understand whether or not the AUROC is fit for clinical practice, we provide additional results on the sensitivity and specificity of our parsimonious model across different cut-off values S9 File, and we leave this choice to clinical judgment.

We also investigated the relevance of the features identified by the SHAP analysis in the original model. The top ten features that we used to develop the parsimonious models are indeed relevant to urine culture outcomes as supported by clinical evidence. For example, previous diseases of the digestive system and previous diseases of the genitourinary system have a strong correlation with the development of infections in the urine [31–34]. The SHAP analysis also revealed that previous ultrasound imaging and microbiology procedures are also predictive of the outcome, which may be related to previous infections or general health issues requiring abdominal imaging [35, 36]. Other identified features include sex, age, and selected dipstick results, which have also been shown to be related to urine culture results in previous work [37–39]. Overall, we note that the top ten features were related to patient demographics (2 out of ten), specific previous diagnosis or procedures (4 out of ten), and dipstick results (4 out of ten), which can all be easily collected and/or acquired from a digital electronic health record system. This implies that the parsimonious model can be easily deployed in existing hospital systems considering its low-cost features.

Our study has several strengths. To the best of our knowledge, our work is the first to develop and validate a model for predicting positive urine cultures in the UAE population, whereas all other related models were developed for populations in the United States or Europe [40–43]. We focus on outpatient visits, rather than a specific patient subgroup. However, the generalizability of this work to other outpatient settings should be treated with caution due to differences in patient demographics, phenotypic variation, practice across healthcare systems, and even practice over time within the same institution [44]. This highlights the importance of model validation in external cohorts.

Another strength is that the parsimonious model achieved comparable performance to the original model and significantly better results than the dipstick only model across both colony count labels. The model can be easily deployed at POC for real-time predictions since it uses easily collected features, compared to other studies that use more complex machine learning models or more expensive features such as genetic or blood biomarkers [40, 45]. By using multivariable logistic regression, our model also offers interpretability since clinicians can refer to the model coefficients or SHAP values assigned to each input feature to understand its importance with respect to the model’s predictions.

While our work focuses on predicting urine culture results, we believe that the proposed model can help in various clinical scenarios where timely urine culture results are needed. Aside from helping in the diagnosis of patients presenting with symptoms of UTI, urine cultures are conducted prior to urological and endoscopic procedures, such as the implantation of urologic prosthetics, urogenital biopsies, and active stone interventions to avoid post-operative infectious complications [46–48]. Furthermore, urine cultures are used in the differential diagnosis of patients suspected of bladder cancer [49], as many of the presenting symptoms overlap with UTI. Other specialties that rely on urine cultures include obstetrics and gynecology, where urine cultures are conducted for pregnant women during the first prenatal visit to check for asymptomatic bacteriuria, which often predisposes UTI, and serious kidney infections such as Pyelonephritis [50]. Such information can especially be useful in settings where urine culture is not easily accessible, hence potentially improving resource allocation and clinical workflow efficiency.

We intend for our proposed model to be an additional tool and source of information in the clinical workflow, like other predictive models. Generally, prediction models are expected to provide the most benefit in identifying patients who are at the highest risk or in assisting in circumstances where urine culture is not easily accessible, hence mostly for operational purposes and resource allocation. We note that the implications of a negative or positive prediction may vary depending on the local guidelines, the patient history, presenting complaints in relation to the suspected disease, differential diagnosis, or patient monitoring motivation behind ordering the urine culture. Further studies are required to assess how the model would affect clinical decision-making, such as prospective studies and some lessons are summarized in the work of Kappen et. al [51].

We also acknowledge that our study has several limitations. First, we observe a performance gap between the female and male subgroups when investigating the model’s performance across patient subgroups. This gap has been identified by other clinical studies for urine dipstick tests, where the diagnostic accuracy of urine dipstick has been found to be higher in males than in females [52]. On the other hand, another study observes a higher performance of an XGboost model in the prediction of suspected urinary tract infections in the emergency department within the female subgroup [53]. This suggests that future work can focus on the development of fairer models across females and males [52].

Another limitation of this work is the possible dependency across multiple encounters for the same patient since we have more unique encounters than unique patients. In the future, we plan to investigate mixed effect logistic regression models [54] to account for any dependencies across samples. Despite the simplicity of the logistic regression model, we also did not investigate more complex machine learning approaches that could lead to better performance results, and this is an area of future work. Finally, this is a single-center retrospective study due to the lack of access to other outpatient-based datasets. In the future, we are interested in conducting a multi-center retrospective study, as well as a prospective validation study to assess the model’s performance in a real-world setting.

It is important to acknowledge a related area of research that specifically focuses on the diagnosis of urinary tract infection, such as the work of [42]. We were unable to obtain definitive labels of infection diagnosis due to the absence of data on patients’ presenting symptoms, which are usually required for a confirmed diagnosis. Our model is not comparable to those in related studies since we focus on the prediction of urine culture results. We also did not rely on ICD codes since they are used for billing purposes and hence may be noisy. Considering that we focus on a general outpatient cohort, we believe that our model can still be used for patients with suspected urinary tract infections, although its use should be in accordance with diagnostic stewardship since the reliance on urine cultures results may lead to misdiagnosis and unnecessary antibiotics [3, 55]. Additionally, model transportability across different levels of care facilities will generally rely on the type of decision that needs to be made based on urine culture results, and how fast it needs to be made. This requires further investigations related to implementation science and the role of predictive algorithms within complex decision making frameworks in health and medicine [56].

## Supporting information

S1 File TRIPOD statement. (PNG)

S2 File ICD-10 codes for defining comorbidities. Included ICD codes ranges used to extract comorbidities from previous patient encounters.
(PDF)

S3 File Hyperparameter search. Values considered during the cross-validated hyperparameter search to select the final parameters to train the multi-variate logistic regression models.
(PDF)

S4 File Results using the 10^4^ cut-off threshold. Performance evaluation results on the test set using the 10^4^ cut-off threshold. We report the performance results for the area under the receiver operating characteristic curve (AUROC), area under the precision-recall curve (AUPRC), and calibration slope and intercept. The results are shown for the overall population and patient sub-groups. All results are reported with 95% confidence intervals computed using bootstrapping with 1,000 iterations.
(PDF)

S5 File Input features for parsimonious models and SHAP values. List of included features along with their SHAP values used to determine their inclusion in the parsimonious models.
(PDF)

S6 File Parameters of parsimonious models. Final coefficients for multivariable -logistic regression-based parsimonious models.
(PDF)

S7 File Performance when varying number of features in parsimonious model. Performance in terms of the receiver operating characteristic curve (AUROC), area under the precision-recall curve (AUPRC) when training and testing the parsimonious model with the top x features, where x is iteratively decreased.
(PDF)

S8 File HTML SHAP analysis. This supplementary file is in HTML format and can be used to check feature importance with respect to model predictions via the SHAP analysis.
(HTM)

S9 File Sensitivity and specificity analysis of the parsimonious model. The table shows the confusion matrix with sensitivity, specificity, TN, FP, FN, and TP at different cutoff risk points. The logistic regression model predictions were binarized by adjusting the alerting threshold to achieve approximately x sensitivity on the test set, where x is referred to as “Risk cut points” in the table.
(PDF)
