# Supplementary material for: Development and validation of a parsimonious prediction model for positive urine cultures in outpatient visits
Source: PLOS Digit Health. 2023 Nov 1;2(11):e0000306. doi: 10.1371/journal.pdig.0000306 (PMC10619807; doi:10.1371/journal.pdig.0000306)
Supplement: S2 File — Included ICD codes ranges used to extract comorbidities from previous patient encounters. (PDF) [file pdig.0000306.s002.pdf]

Development and validation of a parsimonious prediction model for positive urine cultures in outpatient visits

Ghadeer O. Ghosheh[1]*, Terrence Lee St John[2],
Pengyu Wang[1], **Vee Nis Ling**[1], **Lelan Orquiola**[2], **Nasir Hayat**[1]†,
Farah E. Shamout[1,‡], **Y. Zaki Almallah**[2,‡]
[1] NYU Abu Dhabi, Abu Dhabi, The United Arab Emirates
[2] Cleveland Clinic Abu Dhabi, Abu Dhabi, The United Arab Emirates
‡ Equal Supervision

July 10, 2023

S2. ICD-10 codes for defining comorbidities

Features of comorbidities were extracted from previous patient encounters by searching for ICD-10 codes related to the comorbidities of interest. The searched ICD-10 code ranges are shown in Table S2.

Table S2. Included ICD codes ranges used to extract comorbidities from previous patient encounters.

Comorbidity	ICD-10 Code Ranges
Diabetes	[E08-E13]
Hypertension	[I10-I15]
Hyperlipidemia	[E78]

*Currently at the University of Oxford.
†Currently at G42.
