# Supplementary material for: Development and validation of a parsimonious prediction model for positive urine cultures in outpatient visits
Source: PLOS Digit Health. 2023 Nov 1;2(11):e0000306. doi: 10.1371/journal.pdig.0000306 (PMC10619807; doi:10.1371/journal.pdig.0000306)
Supplement: S3 File — Values considered during the cross-validated hyperparameter search to select the final parameters to train the multi-variate logistic regression models. (PDF) [file pdig.0000306.s003.pdf]

Development and validation of a parsimonious prediction model for positive urine cultures in outpatient visits

Ghadeer O. Ghosheh[1,*], Terrence Lee St John[2],
Pengyu Wang[1], **Vee Nis Ling**[1], **Lelan Orquiola**[2], **Nasir Hayat**[1,†],
Farah E. Shamout[1,‡], **Y. Zaki Almallah**[2,‡]
[1] NYU Abu Dhabi, Abu Dhabi, The United Arab Emirates
[2] Cleveland Clinic Abu Dhabi, Abu Dhabi, The United Arab Emirates
‡ Equal Supervision

July 10, 2023

S3. Hyperparameter search

The best hyperparameters selected to train the models were chosen using a 5-fold cross-validation randomized search, which we conducted independently for each of the three models (original, parsimonious, and dipstick). The searched values and ranges for each hyperparameter are shown in Table S3.

Table S3. Values considered during the cross-validated hyperparameter search to select the final parameters to train the multi-variate logistic regression models

Hyperparameters	Values
Regularization parameter C	[1.e-04 - 1.e+04]
Max iterations	[40-300]
Penalty	[l2, l1]
Solver	[*sag*, *saga*, *liblinear*]

*Currently at the University of Oxford.
†Currently at G42.
