# Supplementary material for: Development and validation of a parsimonious prediction model for positive urine cultures in outpatient visits
Source: PLOS Digit Health. 2023 Nov 1;2(11):e0000306. doi: 10.1371/journal.pdig.0000306 (PMC10619807; doi:10.1371/journal.pdig.0000306)
Supplement: S4 File — Performance evaluation results on the test set using the 104 cut-off threshold. We report the performance results for the area under the receiver operating characteristic curve (AUROC), area under the precision-recall curve (AUPRC), and calibration slope and intercept. The results are shown for the overall population and patient sub-groups. All results are reported with 95% confidence intervals computed using bootstrapping with 1,000 iterations. (PDF) [file pdig.0000306.s004.pdf]

Development and validation of a parsimonious prediction model for positive urine cultures in outpatient visits

Ghadeer O. Ghosheh[1]*, Terrence Lee St John[2],
Pengyu Wang[1], **Vee Nis Ling**[1], **Lelan Orquiola**[2], **Nasir Hayat**[1]†,
Farah E. Shamout[1,‡], **Y. Zaki Almallah**[2,‡]
[1] NYU Abu Dhabi, Abu Dhabi, The United Arab Emirates
[2] Cleveland Clinic Abu Dhabi, Abu Dhabi, The United Arab Emirates
‡ Equal Supervision

S4. Results using the 10^4 cut-off threshold

Table S4. Performance evaluation results on the test set using the 10^4 cut-off threshold . We report the performance results for the area under the receiver operating characteristic curve (AUROC), area under the precision-recall curve (AUPRC), and calibration slope and intercept. The results are shown for the overall population and patient sub-groups. All results are reported with 95% confidence intervals computed using bootstrapping with 1,000 iterations [**?**].

Population	Result	Original model	Parsimonious model	Dipstick model
Overall population	AUROC	0.794 (0.778, 0.808)	0.792 (0.776, 0.807)	0.739 (0.722, 0.758)
	AUPRC	0.550 (0.519, 0.582)	0.553 (0.522, 0.582)	0.484 (0.454, 0.518)
	Calibration slope	0.980 (0.904, 1.053)	1.004(0.942, 1.061)	0.975 (0.869, 1.080)
	Calibration intercept	0.016(-0.01, 0.040)	0.004 (-0.018, 0.029)	0.023 (-0.016, 0.066)
Females	AUROC	0.721 (0.698, 0.742)	0.719 (0.696, 0.742)	0.710 (0.689, 0.733)
	AUPRC	0.578 (0.540, 0.614)	0.587 (0.555, 0.622)	0.548 (0.515, 0.585)
	Calibration slope	0.909 (0.818, 0.992)	0.990 (0.911, 1.061)	0.987 (0.851, 1.101)
	Calibration intercept	0.052 (0.017, 0.088)	0.026 (-0.007, 0.06)	0.081 (0.028, 0.136)
Males	AUROC	0.809 (0.782, 0.836)	0.799 (0.770, 0.830)	0.756 (0.726, 0.785)
	AUPRC	0.469 (0.414, 0.528)	0.447 (0.389, 0.510)	0.391 (0.336, 0.448)
	Calibration slope	1.029 (0.903, 1.142)	0.912 (0.733, 1.024)	0.831 (0.545, 1.04)
	Calibration intercept	-0.006 (-0.045, 0.038)	0.012 (-0.033, 0.069)	-0.027 (-0.094, 0.056)
¡ 40 years old	AUROC	0.792 (0.763, 0.819)	0.778 (0.749, 0.808)	0.722 (0.687, 0.757)
	AUPRC	0.447 (0.387, 0.515)	0.450 (0.394, 0.518)	0.386 (0.332, 0.451)
	Calibration slope	0.911 (0.661, 1.071)	0.838 (0.61, 1.041)	0.843 (0.6, 1.076)
	Calibration intercept	0.041 (-0.009, 0.108)	0.055 (-0.022, 0.134)	0.051 (-0.021, 0.127)
$\geq$ 40 years old	AUROC	0.790 (0.773, 0.807)	0.792 (0.775, 0.809)	0.747 (0.728, 0.766)
	AUPRC	0.584 (0.551, 0.618)	0.584 (0.553, 0.618)	0.529 (0.494, 0.561)
	Calibration slope	1.012 (0.931, 1.085)	1.041 (0.986, 1.100)	1.034 (0.961, 1.104)
	Calibration intercept	0.004 (-0.022, 0.033)	-0.011 (-0.035, 0.013)	0.006 (-0.029, 0.045)

*Currently at the University of Oxford.
†Currently at G42.
