# Supplementary material for: Development and validation of a parsimonious prediction model for positive urine cultures in outpatient visits
Source: PLOS Digit Health. 2023 Nov 1;2(11):e0000306. doi: 10.1371/journal.pdig.0000306 (PMC10619807; doi:10.1371/journal.pdig.0000306)
Supplement: S5 File — List of included features along with their SHAP values used to determine their inclusion in the parsimonious models. (PDF) [file pdig.0000306.s005.pdf]

# Development and validation of a parsimonious prediction model for positive urine cultures in outpatient visits

Ghadeer O. Ghosheh[1,*], Terrence Lee St John[2],
**Pengyu Wang**[1], **Vee Nis Ling**[1], **Lelan Orquiola**[2], **Nasir Hayat**[1†],
**Farah E. Shamout**[1,‡], **Y. Zaki Almallah**[2,‡]
[1] NYU Abu Dhabi, Abu Dhabi, The United Arab Emirates
[2] Cleveland Clinic Abu Dhabi, Abu Dhabi, The United Arab Emirates
‡ Equal Supervision

## S5. Input features for parsimonious models and SHAP values

The full list of features used to train the original models for each threshold, along with the features' SHAP values. Features with the highest SHAP values for each threshold were then used to train each of the parsimonious models, as shown in Table S5.

*Currently at the University of Oxford.
†Currently at G42.

**Table S5.** List of included features along with their SHAP values used to determine their inclusion in the parsimonious models.

| | SHAP values | | Parsimonious Model | |
|---|---|---|---|---|
| **Feature Name** | $\mathbf{10^5}$ | $\mathbf{10^4}$ | $\mathbf{10^5}$ | $\mathbf{10^4}$ |
| leukocyte esterase negative | 0.370 | 0.299 | ✓ | ✓ |
| male | 0.368 | 0.377 | ✓ | ✓ |
| age | 0.244 | 0.210 | ✓ | ✓ |
| hemoglobin negative | 0.219 | 0.131 | ✓ | ✓ |
| previous diseases of the digestive system | 0.196 | 0.133 | ✓ | ✓ |
| nitrite positive | 0.183 | 0.149 | ✓ | ✓ |
| leukocyte esterase 3+ | 0.167 | 0.150 | ✓ | ✓ |
| previous microbiology - general orderables | 0.130 | 0.114 | ✓ | ✓ |
| previous diseases of the genitourinary system | 0.113 | 0.083 | ✓ | ✓ |
| previous imgaging ultrasound orderables | 0.111 | 0.069 | ✓ | |
| previous imgaging ct orderables | 0.085 | 0.037 | | |
| previous symptoms, signs, & abnormal clinical laboratory findings | 0.084 | 0.057 | | |
| previous diseases of the musculoskeletal system and connective tissue | 0.078 | 0.035 | | |
| poct orderables - device | 0.071 | 0.042 | | |
| previous diseases of the circulatory system | 0.068 | 0.062 | | |
| urine orderables | 0.065 | 0.075 | | ✓ |
| pft orderables | 0.053 | 0.032 | | |
| leukocyte esterase trace | 0.051 | 0.061 | | |
| leukocyte esterase 2+ | 0.050 | 0.043 | | |
| previous diseases of the nervous system | 0.047 | 0.037 | | |
| diastolic blood pressure | 0.045 | 0.067 | | |
| factors influencing health status and contact with health services | 0.045 | 0.018 | | |
| previous diseases of the respiratory system | 0.040 | 0.050 | | |
| previous diseases of the skin and subcutaneous tissue | 0.039 | 0.029 | | |
| previous cv ecg / ekg orderables | 0.038 | 0.032 | | |
| hemoglobin trace | 0.038 | 0.008 | | |
| prev diabetes | 0.037 | 0.017 | | |
| previous procedure/minor surgical orderables | 0.037 | 0.029 | | |
| previous imaging fluoroscopy orderables | 0.027 | 0.027 | | |
| previous lab blood orderables | 0.027 | 0.013 | | |
| previous pathology/cytology orderables | 0.024 | 0.021 | | |
| leukocyte esterase 1+ | 0.022 | 0.011 | | |
| previous imaging MRI orderables | 0.022 | 0.035 | | |
| hemoglobin 3+ | 0.019 | 0.023 | | |
| hemoglobin 1+ | 0.018 | 0.016 | | |
| previous lab chemistry orderables | 0.011 | 0.037 | | |
| respiratory rate | 0.010 | 0.056 | | |
| prev hypertension | 0.008 | 0.002 | | |
| hla lab orderables | 0.007 | 0.002 | | |
| systolic blood pressure | 0.007 | 0.025 | | |
| temperature | 0.006 | 0.008 | | |
| pulse | 0.006 | 0.017 | | |
| previous adt orderables | 0.006 | 0.003 | | |
| previous diseases of the blood and blood-forming organs | 0.005 | 0.005 | | |
| previous neoplasms | 0.005 | 0.005 | | |
| previous diseases of the eye and adnexa | 0.005 | 0.005 | | |
| previous diseases of the ear and mastoid process | 0.005 | 0.005 | | |
| previous GI procedure orderables | 0.004 | 0.001 | | |
| previous ophthalmology zeiss orderables | 0.004 | 0.007 | | |
| previous cv echo orderables | 0.003 | 0.007 | | |
| mental, behavioral and neurodevelopmental disorders | 0.003 | 0.008 | | |
| oxygen saturation | 0.003 | 0.003 | | |
| previous diagnostic imaging/x-ray orderables | 0.003 | 0.014 | | |
| blood bank product orderables | 0.003 | 0.002 | | |
| img dexa orderables | 0.002 | 0.002 | | |
| previous endocrine, nutritional and metabolic previous diseases | 0.002 | 0.010 | | |
| previous outpatient referral orderables | 0.002 | 0.001 | | |
| previous general surgical orderables | 0.001 | 0.004 | | |
| prev hyperlipemia | 0.001 | 0.007 | | |
| previous injury, poisoning, and certain other consequences of external causes | 0.001 | 0.010 | | |
| hemoglobin 4+ | 0.001 | 0.002 | | |
| previous congenital malformations, deformations and chromosomal abnormalities | 0.001 | 0.005 | | |
| previous genetic testing | 0.001 | 0.004 | | |
| previous certain infections and parasitic previous diseases | 0.001 | 0.012 | | |
| previous blood bank test orderables | 0.001 | 0.000 | | |
| cancer | 0.000 | 0.000 | | |
| previous neurology orderables | 0.000 | 0.001 | | |
| hemoglobin 2+ | 0.000 | 0.004 | | |
| previous imaging NM orderables | 0.000 | 0.002 | | |
| previous imaging mammography orderables | 0.000 | 0.000 | | |
| previous ophthalmology services orderables | 0.000 | 0.000 | | |
| previous body fluids and stools orderables | 0.000 | 0.002 | | |
| previous pregnancy, childbirth, and puerperium | 0.000 | 0.001 | | |
| previous ent orderables | 0.000 | 0.000 | | |
| previous oncbcn communication | 0.000 | 0.000 | | |
| previous certain conditions originating in the perinatal period | 0.000 | 0.000 | | |
| previous external causes of morbidity | 0.000 | 0.000 | | |
| previous core measures orderables | 0.000 | 0.000 | | |
| previous visit type linked ref orders | 0.000 | 0.000 | | |
| previous codes for special purposes | 0.000 | 0.000 | | |
