# Supplementary material for: Development and validation of a parsimonious prediction model for positive urine cultures in outpatient visits
Source: PLOS Digit Health. 2023 Nov 1;2(11):e0000306. doi: 10.1371/journal.pdig.0000306 (PMC10619807; doi:10.1371/journal.pdig.0000306)
Supplement: S6 File — Final coefficients for multivariable -logistic regression-based parsimonious models. (PDF) [file pdig.0000306.s006.pdf]

Development and validation of a parsimonious prediction model for positive urine cultures in outpatient visits

Ghadeer O. Ghosheh[1,*], Terrence Lee St John[2],
Pengyu Wang[1], **Vee Nis Ling**[1], **Lelan Orquiola**[2], **Nasir Hayat**[1,†],
Farah E. Shamout[1,‡], **Y. Zaki Almallah**[2,‡]
[1] NYU Abu Dhabi, Abu Dhabi, The United Arab Emirates
[2] Cleveland Clinic Abu Dhabi, Abu Dhabi, The United Arab Emirates
‡ Equal Supervision

S6. Parameters of parsimonious models

Two parsimonious models were trained using the top predictive features for each threshold, 10^5 and 10^4 respectively. The logistic regression coefficients for each of the parsimonious models are shown in Table S6.

Table S6. Final coefficients for multivariable -logistic regression-based parsimonious models

	$\geq 10^5$ label	
**Coefficients**	leukocyte esterase negative	-0.55
	male	-0.64
	age	0.02
	hemoglobin negative	0.37
	previous diseases of the digestive system	-0.42
	nitrite positive	2.80
	leukocyte esterase 3+	1.05
	previous procedure microbiology general orderables	0.33
	previous diseases of the genitourinary system	0.27
	previous procedure-img us orderables	0.36
**Intercept**		-3.24
	$\geq 10^4$ label	
**Coefficients**	male	-0.73
	leukocyte esterase negative	-0.56
	age	0.02
	leukocyte esterase 3+	0.93
	nitrite positive	2.43
	previous diseases of the digestive system	-0.26
	hemoglobin negative	0.22
	previous procedure microbiology general orderables	0.28
	previous diseases of the genitourinary system	0.27
	previous procedure urine orderables	0.19
**Intercept**		-2.23

*Currently at the University of Oxford.
†Currently at G42.
