# Supplementary material for: Development and validation of a parsimonious prediction model for positive urine cultures in outpatient visits
Source: PLOS Digit Health. 2023 Nov 1;2(11):e0000306. doi: 10.1371/journal.pdig.0000306 (PMC10619807; doi:10.1371/journal.pdig.0000306)
Supplement: S7 File — Performance in terms of the receiver operating characteristic curve (AUROC), area under the precision-recall curve (AUPRC) when training and testing the parsimonious model with the top x features, where x is iteratively decreased. (PDF) [file pdig.0000306.s007.pdf]

Development and validation of a parsimonious prediction model for positive urine cultures in outpatient visits

Ghadeer O. Ghosheh[1]*, Terrence Lee St John[2],
Pengyu Wang[1], **Vee Nis Ling**[1], **Lelan Orquiola**[2], **Nasir Hayat**[1]†,
Farah E. Shamout[1,‡], **Y. Zaki Almallah**[2,‡]
[1] NYU Abu Dhabi, Abu Dhabi, The United Arab Emirates
[2] Cleveland Clinic Abu Dhabi, Abu Dhabi, The United Arab Emirates
‡ Equal Supervision

S7. Number of features in parsimonious model

We experiment with reducing the number of features used to train the parsimonious model on the 10^5 threshold. The results for all models are reported in terms of the receiver operating characteristic curve (AUROC), area under the precision-recall curve (AUPRC), as shown in Table S7.

Table S7. Performance in terms of the receiver operating characteristic curve (AUROC), area under the precision-recall curve (AUPRC) when training and testing the parsimonious model with the top x features, where x is iteratively decreased.

**Input features**	**AUROC**	**AUPRC**
10	0.828 (0.812, 0.844)	0.550 (0.516, 0.588)
9	0.826 (0.809, 0.841)	0.547 (0.510, 0.583)
8	0.825 (0.809, 0.840)	0.548 (0.513, 0.582)
7	0.821 (0.804, 0.838)	0.544 (0.508, 0.581)
6	0.816 (0.798, 0.833)	0.537 (0.501, 0.573)
5	0.757 (0.739, 0.776)	0.371 (0.338, 0.409)
4	0.758 (0.739, 0.776)	0.370 (0.338, 0.405)
3	0.757 (0.739, 0.777)	0.366 (0.331, 0.404)
2	0.749 (0.732, 0.765)	0.281 (0.259, 0.305)
1	0.700 (0.682, 0.718)	0.246 (0.227, 0.267)

*Currently at the University of Oxford.
†Currently at G42.
