# Supplementary material for: Development and validation of a parsimonious prediction model for positive urine cultures in outpatient visits
Source: PLOS Digit Health. 2023 Nov 1;2(11):e0000306. doi: 10.1371/journal.pdig.0000306 (PMC10619807; doi:10.1371/journal.pdig.0000306)
Supplement: S8 File — This supplementary file is in HTML format and can be used to check feature importance with respect to model predictions via the SHAP analysis. (HTM) [file pdig.0000306.s008.htm]

 

**Visualization omitted, Javascript library not loaded!**  
Have you run `initjs()` in this notebook? If this notebook was from another
user you must also trust this notebook (File -> Trust notebook). If you are viewing
this notebook on github the Javascript has been stripped for security. If you are using
JupyterLab this error is because a JupyterLab extension has not yet been written.
