# Supplementary material for: Development and validation of a parsimonious prediction model for positive urine cultures in outpatient visits
Source: PLOS Digit Health. 2023 Nov 1;2(11):e0000306. doi: 10.1371/journal.pdig.0000306 (PMC10619807; doi:10.1371/journal.pdig.0000306)
Supplement: S9 File — The table shows the confusion matrix with sensitivity, specificity, TN, FP, FN, and TP at different cutoff risk points. The logistic regression model predictions were binarized by adjusting the alerting threshold to achieve approximately x sensitivity on the test set, where x is referred to as “Risk cut points” in the table. (PDF) [file pdig.0000306.s009.pdf]

# Development and validation of a parsimonious prediction model for positive urine cultures in outpatient visits

Ghadeer O. Ghosheh[1]*, Terrence Lee St John[2],
**Pengyu Wang**[1], **Vee Nis Ling**[1], **Lelan Orquiola**[2], **Nasir Hayat**[1]†,
**Farah E. Shamout**[1,‡], **Y. Zaki Almallah**[2,‡]
[1] NYU Abu Dhabi, Abu Dhabi, The United Arab Emirates
[2] Cleveland Clinic Abu Dhabi, Abu Dhabi, The United Arab Emirates
‡ Equal Supervision

## S9. Sensitivity and specificity analysis of the parsimonious model

We conduct a sensitivity and specificity analysis for our parsimonious model at various risk cut points. The logistic regression model predictions were converted to binary by adjusting the alerting threshold to achieve approximately x sensitivity on the test set, where x is referred to as "Risk cut points" in the Table S9.

**Table S9.** The table shows the confusion matrix with sensitivity, specificity, TN, FP, FN, and TP at different cutoff risk points. The logistic regression model predictions were binarized by adjusting the alerting threshold to achieve approximately x sensitivity on the test set, where x is referred to as "Risk cut points" in the table.

| Cut-off | Sensitivity | Specificity | TN | FP | FN | TP |
|---|---|---|---|---|---|---|
| 0.1 | 0.764 | 0.702 | 312 | 1358 | 181 | 588 |
| 0.2 | 0.537 | 0.911 | 4161 | 409 | 354 | 415 |
| 0.3 | 0.415 | 0.961 | 4391 | 179 | 450 | 319 |
| 0.4 | 0.354 | 0.979 | 4475 | 95 | 498 | 271 |
| 0.5 | 0.298 | 0.985 | 4500 | 70 | 540 | 229 |
| 0.6 | 0.242 | 0.989 | 4522 | 48 | 583 | 186 |
| 0.7 | 0.183 | 0.992 | 4535 | 35 | 628 | 141 |
| 0.8 | 0.140 | 0.996 | 4451 | 19 | 661 | 108 |
| 0.9 | 0.040 | 0.999 | 4567 | 3 | 738 | 31 |

*Currently at the University of Oxford.
†Currently at G42.
